# Interlocking pentagons task and visuospatial deficit in parkinsonian syndrome: a quantitative approach

**DOI:** 10.1007/s00415-026-14163-8

**Published:** 2026-09-28

**Authors:** Giulia Donzuso, F. Cilio, E. Ferrante, C. D’Agate, G. Fazzina, C. E. Cicero, G. Mostile, A. Nicoletti, M. Zappia

**Affiliations:** 1 Department of Medical, Surgical Sciences and Advanced Technologies “G.F. Ingrassia”, University of Catania, Via Santa Sofia 78, 95123 Catania, Italy; 2 Oasi Research Institute - IRCCS, Troina, Italy; 3 AOU Policlinico “G. Rodolico-San Marco”, Catania, Italy

**Keywords:** Parkinson’s disease, Atypical parkinsonism, Visuoconstructive function, Differential diagnosis, MMSE pentagon

## Abstract

**Objectives:**

Visuo-spatial impairment is a common feature in parkinsonian disorders and differentiating idiopathic Parkinson’s disease (PD) from atypical parkinsonian syndromes (APS), i.e. progressive supranuclear palsy (PSP), multiple system atrophy (MSA), and corticobasal degeneration (CBD), remains a major clinical challenge. The Qualitative Scoring Pentagon Test (QSPT), derived from the MMSE pentagon-copying item, provides quantitative visuo-constructive assessment but has not been systematically evaluated as a differential marker in parkinsonism. Aim of the study was to assess the discriminative value of the QSPT total score and sub-items across parkinsonian syndromes, and to examine associations with neuropsychological tests, motor severity, and MRI morphometric indices.

**Methods:**

Patients with PD and APS were enrolled and underwent clinical and neuropsychological evaluations including MMSE, Frontal Assessment Battery (FAB), Trail Making Test (TMT-A), and MDS-Unified Parkinson’s Disease Rating Scale-Section III (MDS-UPDRS-III). A group of healthy controls (HC) were also enrolled performing MMSE. Pentagon drawings were scored quantitatively (QSPT). When available, MRI morphometric features were collected. Statistical analysis were performed to assess associations between QSPT, cognitive and clinical features, and to evaluate differences between patients and HC and across patients’ groups.

**Results:**

One-hundred and forty patients (PD=91, PSP=23, MSA=17, CBD=9) and 92 HC were enrolled. QSPT differed significantly considering HC performances (12.7 ± 0.5) versus patients groups, and across groups (p<0.001), with PD scoring the highest (11.5 ± 2.1) and CBD the lowest score (7.7 ± 4.4). PD differed from PSP (p<0.001), CBD (p<0.001) and MSA (p=0.040). Rotation and number-of-angles sub-items were the most discriminating features. QSPT significantly correlated with MMSE, FAB, TMT-A, and MRPI (all p<0.01). Multivariate models demonstrated that QSPT was independently associated with APS (OR=0.620, 95%CI 0.469–0.820, p<0.001). A QSPT score ≤ 8 points discriminated PD from APS with an AUC of 0.764 and a specificity of 92.3%, and ≤ 11 points discriminated synucleinopathies from tauopathies with a higher AUC (0.804).

**Conclusion:**

This study demonstrates that quantitative scoring of the MMSE pentagon-copying item could differentiate PD from APS. The presence of association between QSPT and MRI brainstem morphometry could also reflect a structural association, specifically identifying tauopathies. QSPT could be a simple and supportive clinical tool, embedded within the MMSE that could differentiates patients from HC and PD from APS.

**Supplementary Information:**

The online version contains supplementary material available at https://doi.org/10.1007/s00415-026-14163-8.

## Introduction

Parkinson’s disease (PD) is the second most frequent neurodegenerative disorder [1] and its early clinical distinction from atypical parkinsonian syndromes (APS)—progressive supranuclear palsy (PSP), multiple system atrophy (MSA), and corticobasal degeneration (CBD)—is among the most demanding diagnostic challenges in movement disorders [2, 3]. Misdiagnosis of these conditions may lead to significant clinical consequences, including inappropriate dopaminergic therapy, misinformed prognosis, and exclusion from targeted trials [4].

Cognitive impairment in APS is early, prominent, and diagnostically informative [5]. CBD and PSP patients show marked frontal-executive and visuoconstructive deficits due to parietal and frontal-cortical involvement [5, 6], while MSA may present with relatively preserved cognition, although frontal-executive dysfunction is the most common presentation [7]. Patients with PD present a more heterogeneous cognitive pattern with prevalent executive dysfunction, even at an early stage [8]. Visuoconstructive ability, assessed through simple copying tasks, reflects a network covering parieto-occipital, frontal, and basal ganglia circuits, all differentially affected across parkinsonian syndromes [9, 10].

In this context, the pentagon copying item of the Mini-Mental State Examination (MMSE), traditionally scored as pass/fail [11], could be used as a practical tool for identifying cognitive and visuospatial decline in both PD and APS [12]. Furthermore, it can be quantitatively rescored using the Qualitative Scoring Pentagon Test (QSPT) developed by Caffarra et al. [13]. This method has been validated as a discriminator between dementia with Lewy bodies and Alzheimer’s disease [13, 14], and a more detailed digital quantification approach has recently been proposed [15]. Brain MRI correlates of the pentagon item have been detected in posterior regions, confirming the role of these areas in the modulation of visuospatial functions [16].

On this background, the aim of the present study is to compare QSPT scores across PD and APS patients, and to evaluate possible neuropsychological and MRI associations, proposing QSPT as a potentially useful differential clinical biomarker.

## Materials and methods

### Study population

We conducted a retrospective cross-sectional study enrolling patients with PD and APS attending the Neurology Clinic of the University of Catania. Clinical diagnoses were established by experienced movement disorder neurologists applying current consensus criteria [6, 17–19]. Patients with MMSE <24 or inability to attempt the pentagon task were excluded. Additionally, 92 healthy controls (HC) without neurological or psychiatric disease, recruited among partner or caregiver of patients, were enrolled as normative reference group. The collection of data was approved by our local Ethical Committee Catania 1 (Code 316/2015) and all participants provided informed consent in accordance with the Declaration of Helsinki.

### Neuropsychological and motor assessment

All patients underwent a standardized neuropsychological and clinical evaluation including MMSE with quantitative pentagon scoring QSPT, Frontal Assessment Battery (FAB), Trail Making Test Part A (TMT-A, seconds), MDS-Unified Parkinson’s Disease Rating Scale-Section III (MDS-UPDRS-III) for motor severity. Current levodopa equivalent daily dose (LEDD) has also been calculated [20].

### QSPT scoring

**Fig. 1:**
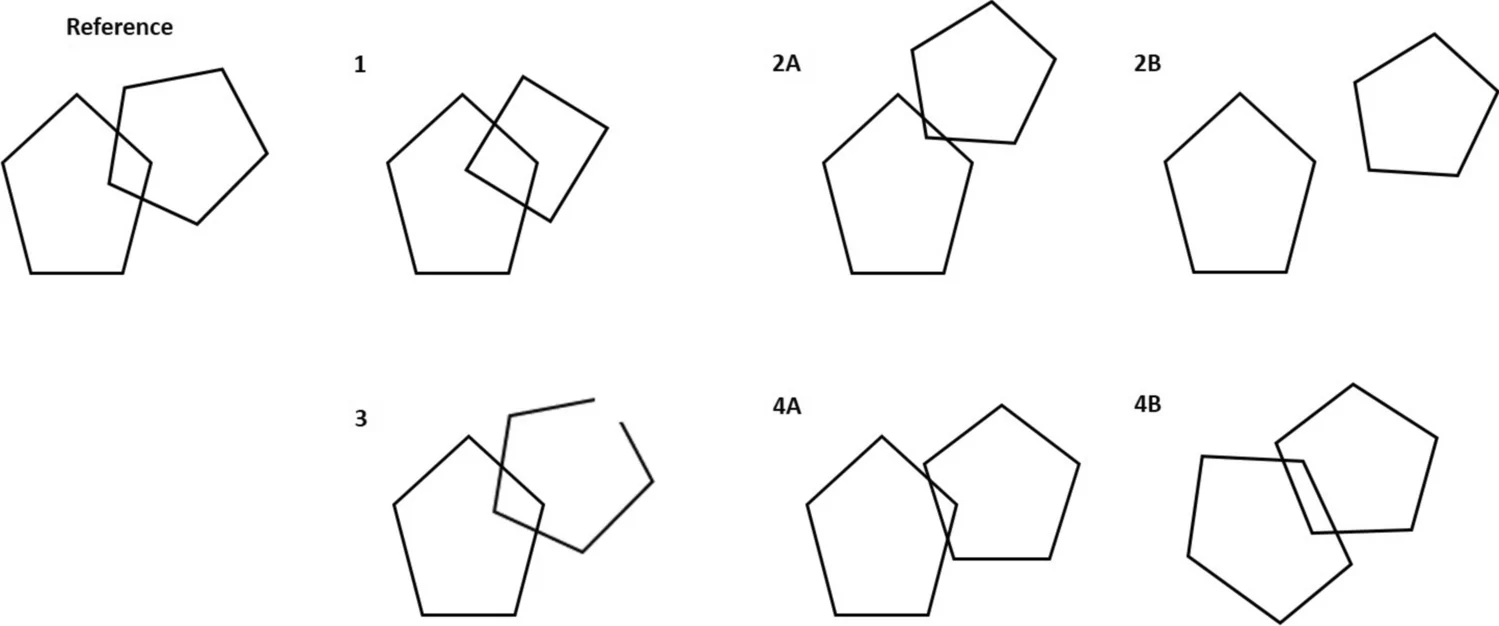
QSPT scoring example. 1. Number of angles (0–4): 3 points; 2. Intersection accuracy (0–4), 2A: 3 points, 2B: 0 points; 3. Open closure (0–2): 1 point; 4. Rotation (0–2), 4A: 1 point, B: 0 points. *QSPT* Qualitative Scoring Pentagon Test. See text for reference

Pentagon drawings were scored using the QSPT method proposed by Caffarra et al. [13]. Briefly, QSPT total score ranges from 0 to 13 and the quantitative score was assigned as following, considering sub-scores to the included sub-items: (1) Number of angles, considering the correct reproduction of the 10 internal angles: score range from 0 to 4; (2) Intersection accuracy, considering the fidelity of the overlapping zone; score range from 0 to 4; (3) Open closure, with the degree to which figure contours are closed: score range from 0 to 2; (4) Rotation, considering the angular displacement from the model: score range from 0 to 2; (5) Closing-in: presence of the closing-in phenomenon, 0 point, absent 1 point (Fig. 1). All drawings were scored by two trained raters blinded to diagnosis.

### MRI morphometry

When available, brain MRI was acquired in order to obtain morphometric measurements and Magnetic Resonance Parkinsonism Index (MRPI), as previously described [21]. Briefly, using sagittal T1-3D weighted sequences pons and midbrain area (mm^2^), middle cerebellar peduncle (MCP) and superior cerebellar peduncle (SCP) width (mm) were obtained, and the MRPI [(pons area/midbrain area) × (MCP width/SCP width)], was calculated. All measurements were performed by an expert rater blinded to clinical data, according to established protocols.

### Statistical analysis

Analyses were performed in Python (scipy v1.10, sklearn v1.2). All variables were assessed for normality using the Shapiro–Wilk test; non-normal distributions were confirmed for all continuous variables. Group comparisons used the Kruskal–Wallis test and pairwise Mann–Whitney U tests. Spearman’s rank correlation and logistic regression adjusted for LEDD, MDS-UPDRS-ME and disease duration was applied for association analysis. Finally, receiver operating characteristic (ROC) analysis with AUC assessed QSPT discriminative accuracy for PD vs APS; the optimal cutoff was selected by maximising the Youden index. Statistical significance was set at p<0.05. An additional ROC analysis considering synucleinopathies (PD and MSA) *versus* tauopathies (PSP and CBD) was performed.

## Results

### Sample characteristics

One-hundred and forty patients (91 PD, 23 PSP, 17 MSA, and 9 CBD) and 92 HC were enrolled. HC were matched to the PD patient cohort for age and MMSE total score. Demographic, clinical, and neuropsychological characteristics of patient groups are reported in Table 1. Gender was equally distributed in the whole sample (p=0.912), while groups differed significantly in age (PSP and CBD patients were older than PD and MSA, p=0.002), and disease duration (PD had the longest disease duration, p=0.028). Considering cognitive performances, mean MMSE score was significantly different across groups (p=0.015), although in the normal range, together with FAB total score (p<0.001) and TMT-A time (p<0.001), with PSP and CBD patients performing worse than PD and MSA. MDS-UPDRS-III score and LEDD did not differ significantly across groups (p=0.110 and p=0.475, respectively). Considering MRI morphometry (106 MRI data were available), PSP patients showed a markedly elevated MRPI (15.5 ± 3.3), while patients with MSA exhibited the lowest MRPI values (7.5 ± 3.6) (Table 1).

**Table 1:** Demographic and clinical characteristics by diagnostic group

	HC (n=92)	PD (n=91)	MSA (n=17)	PSP (n=23)	CBD (n=9)	p-value*
Age, years	64.5 ± 9.6	64.2 ± 9.9	64.6 ± 6.6	71.4 ± 9.6	71.5 ± 6.5	**0.002**
Gender, male (%)	34 (37.0)	53 (58.2)	9 (52.9)	12 (52.2)	5 (55.6)	0.912
Disease duration, years	/	6.0 ± 4.1	3.2 ± 1.5	4.5 ± 2.5	3.2 ± 1.8	**0.028**
MMSE, score	27.9 ± 1.4	28.0 ± 1.3	28.4 ± 1.5	27.5 ± 1.6	26.6 ± 1.6	**0.015**
FAB, score	/	15.1 ± 2.3	15.9 ± 2.2	12.2 ± 3.8	12.1 ± 3.7	**<0.001**
UPDRS-ME, score	/	38.2 ± 15.3	47.5 ± 20.8	44.9 ± 15.4	32.4 ± 10.9	0.110
TMT-A, sec	/	56.9 ± 30.5	88.2 ± 48.8	136.2 ± 68.8	93.3 ± 75.9	**<0.001**
LEDD	/	530.9 ± 268.6	408.3 ± 192.6	454.2 ± 240.5	345.0 ± 41.1	0.475
		**PD (n=62)**	**MSA (n=13)**	**PSP (n=23)**	**CBD (n=8)**	
MRPI	/	10.0 ± 1.6	7.5 ± 3.6	15.5 ± 3.3	11.8 ± 1.0	<0.001

Data shown as mean ± SD. *p-values from Kruskal–Wallis (continuous) or chi-squared (categorical) across APS groups. *MMSE* Mini-Mental State Examination, *FAB* Frontal Assessment Battery, *UPDRS-ME* Unified Parkinson’s Disease Rating Scale Motor Examination, *TMT-A* Trail Making Test-A, *LEDD* levodopa equivalent daily dose, *MRPI* magnetic resonance parkinsonism index. p<0.05

### QSPT total score, correlations, and diagnostic accuracy

**Fig. 2:**
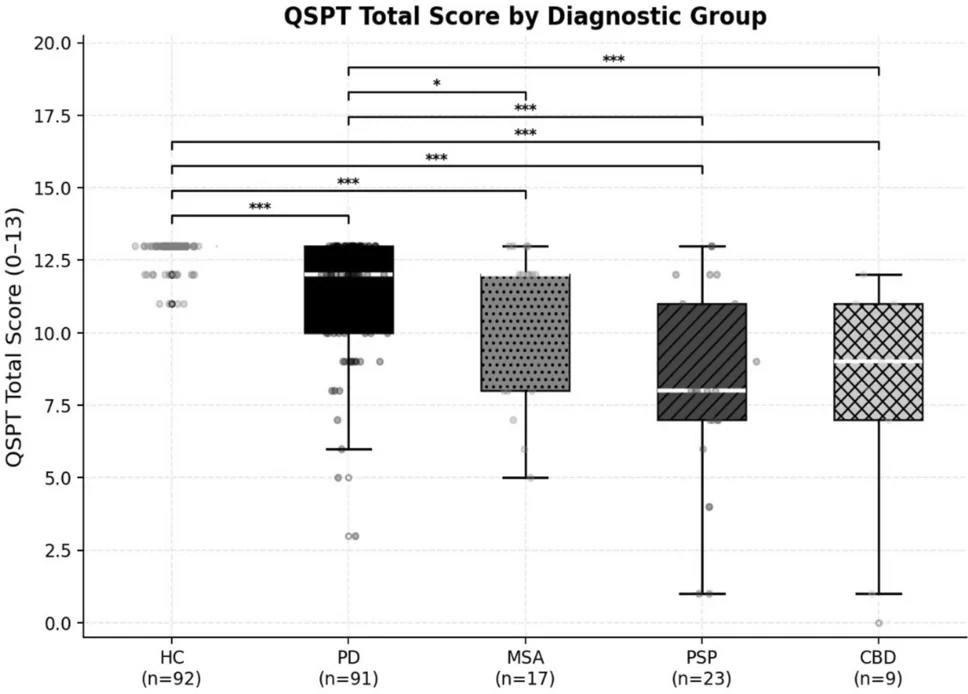
Distribution of QSPT total scores by diagnostic group, including healthy controls (HC). Boxes represent the interquartile range; horizontal lines denote medians; dots represent individual data points. Significance bars indicate pairwise comparisons (***p<0.001; *p<0.05). *QSPT* Qualitative Scoring Pentagon Test, *HC* healthy controls, *PD* Parkinson’s disease, *PSP* progressive supranuclear palsy, *MSA* multiple system atrophy, *CBD* corticobasal degeneration

Table 2 reported QSPT total and sub-items scores across groups. HC achieved highest scores (12.74 ± 0.55), and QSPT was significantly lower in all parkinsonian groups (all p<0.001), with the largest effect size observed for HC vs CBD (r=−0.959) and the smallest for HC vs PD (r=−0.370). Among patients, QSPT total score showed statistically significant differences (p<0.001), with PD patients achieving the highest mean score (11.5 ± 2.1), while CBD patients scored the lowest (7.7 ± 4.4) (Table 2 and Fig. 2). Post-hoc analyses revealed significant differences between PD and PSP (p<0.001), PD and CBD (p<0.001), and PD and MSA (p=0.040). PSP and MSA patients also differed significantly (p=0.042). No significant differences were observed between PSP and CBD. Sub-item scores followed the same pattern with PD patients performing better than the APS subgroup in almost all sub-items, especially vs PSP and CBD subgroups (Table S1 and Figure S1).

**Table 2:** QSPT total and sub-item scores

Variable	HC	PD	MSA	PSP	CBD	p-value (patients)
QSPT total score (0–13)	12.74 ± 0.55	11.5 ± 2.1	10.3 ± 2.8	8.1 ± 3.4	7.7 ± 4.4	**<0.001**
Number of angles (0–4)	3.96 ± 0.25	3.64 ± 0.72	3.12 ± 1.17	2.48 ± 1.44	2.44 ± 1.74	**<0.001**
Intersection accuracy (0–4)	3.92 ± 0.27	3.53 ± 0.66	3.35 ± 0.61	2.48 ± 1.34	2.44 ± 1.42	**<0.001**
Open closure (0–2)	1.87 ± 0.40	1.95 ± 0.23	1.88 ± 0.33	1.78 ± 0.60	1.44 ± 0.88	**0.030**
Rotation (0–2)	2.00 ± 0.00	1.42 ± 0.87	1.00 ± 0.94	0.52 ± 0.79	0.56 ± 0.73	**<0.001**
Closing in (0–1) present, n (%)	0 (0)	0 (0)	1 (5.9)	3 (13)	2 (22.2)	**<0.001**

Data shown as mean ± SD, and frequency of closing in sub-item; post-hoc Mann–Whitney U, PD vs PSP, PD vs MSA, PD vs CBD, and Fisher’s exact test for the closing-in sub-item. *QSPT* Qualitative Scoring Pentagon Test, *PD* Parkinson’s disease, *PSP* progressive supranuclear palsy, *MSA* multisystem atrophy, *CBD* corticobasal degeneration. p<0.05

**Fig. 3:**
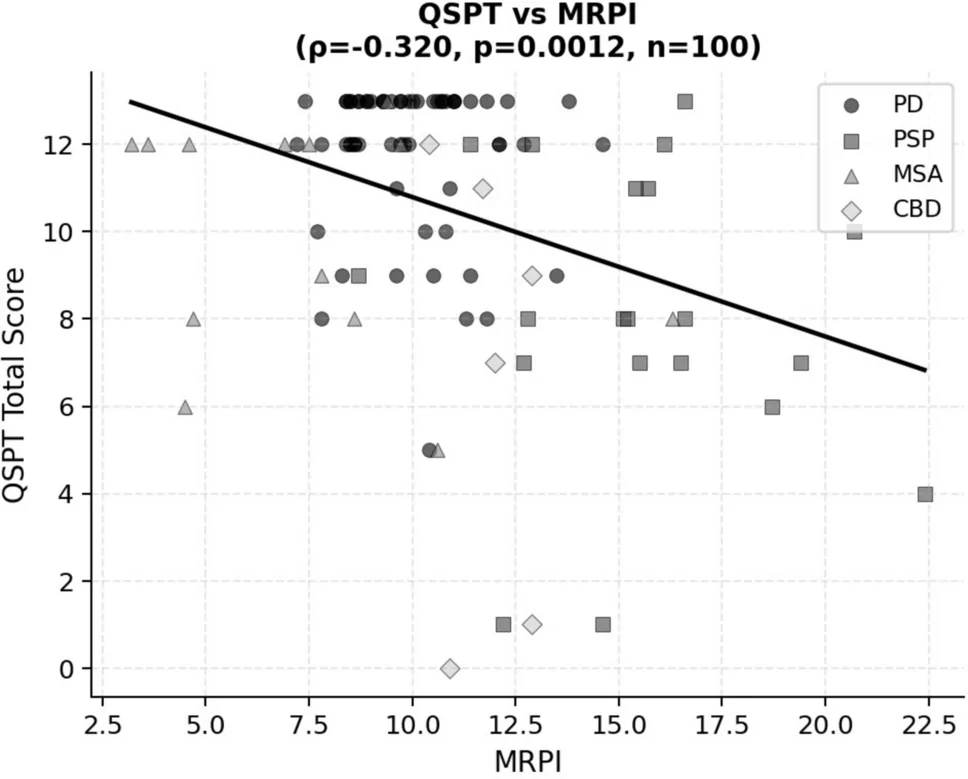
Scatter plot of QSPT total score against MRPI across all patients. Marker shapes distinguish diagnostic groups (circles: PD; squares: PSP; triangles: MSA; diamonds: CBD). Regression line indicates the overall linear trend. *QSPT* Qualitative Scoring Pentagon Test, *MRPI* magnetic resonance parkinsonism index, *PD* Parkinson’s disease, *PSP* progressive supranuclear palsy, *MSA* multiple system atrophy, *CBD* corticobasal degeneration

Considering MRI, QSPT significantly correlated with MRPI across the whole patient sample (n=106; r=−0.320, p=0.001; Fig. 3). Additionally, QSPT was associated with MMSE (r=0.293, p=0.003), FAB (r=0.355, p<0.001), TMT-A (r=−0.481, p<0.001), whereas there were no correlations with disease duration and LEDD.

**Fig. 4:**
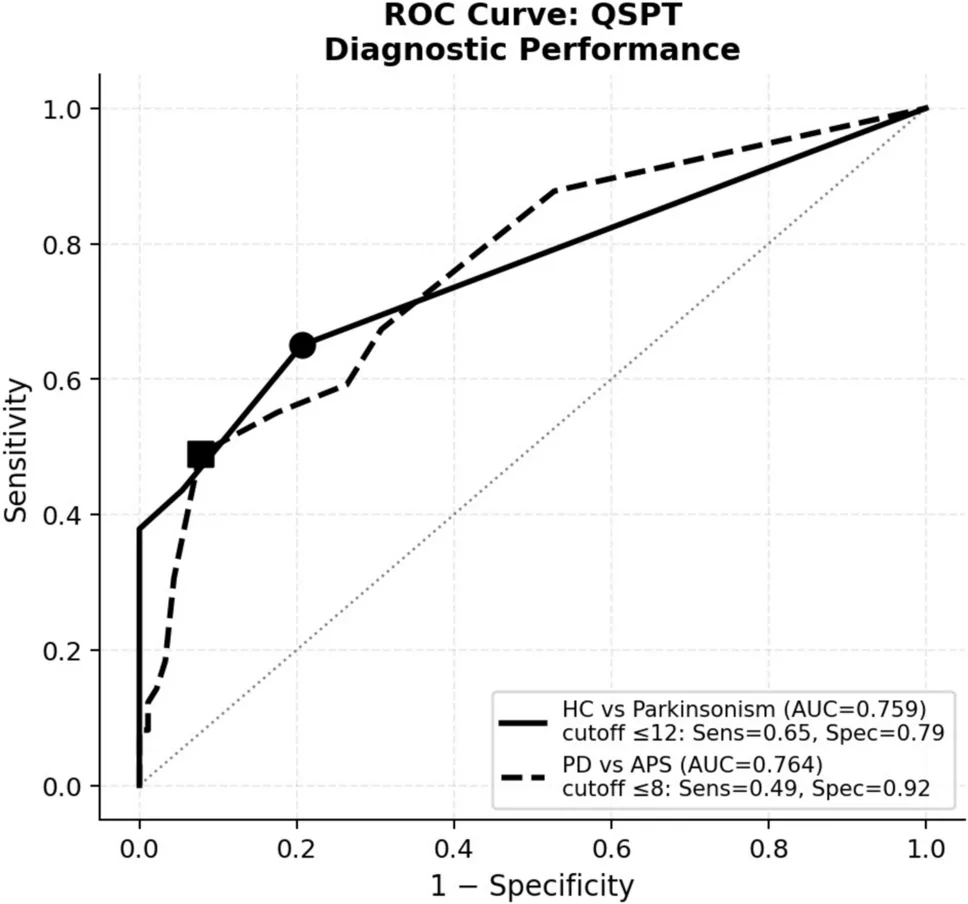
ROC curves for QSPT total score. Solid line: HC vs all parkinsonian patients (AUC=0.759; optimal cutoff ≤12, sensitivity=0.65, specificity=0.79). Dashed line: PD vs atypical parkinsonism (AUC=0.764; optimal cutoff ≤8, sensitivity=0.49, specificity=0.92). = *ROC* receiver operating characteristic, *AUC* area under the curve, *QSPT* Qualitative Scoring Pentagon Test

Logistic regression analysis adjusted for MDS-UPDRS-ME, LEDD and disease duration showed a significant and stronger association between QSPT and APS (OR=0.620, 95%CI 0.469–0.820, p<0.001). Finally, ROC analysis for HC vs all parkinsonian patients yielded an AUC of 0.759 (95%CI 0.693–0.825), with optimal cutoff ≤12 providing sensitivity of 65.0% and specificity of 79.3%, and ROC analysis for QSPT total score in discriminating PD from APS yielded an AUC of 0.764 (95%CI 0.684–0.844) (Fig. 4). The optimal cutoff of ≤8 provided sensitivity of 49.0% and specificity of 92.3% (Youden index=0.413).

When considering synucleinopathies *versus* tauopathies, QSPT total score was statistically significant (p<0.001), and an AUC of 0.804 with an optimal cutoff of ≤11 was achieved obtaining a sensitivity of 81.2% and specificity of 67.6% (figure S2).

## Discussion

This study demonstrates that quantitative scoring of the MMSE pentagon copying item (QSPT) significantly discriminates PD from APS. Additionally, the significant correlation between QSPT and neuropsychological tests including FAB and TMT-A, and MRPI suggested that this simple task could provide insight on cognitive involvement of frontal-executive network and brainstem structures especially in APS patients.

In this context, HC demonstrated near-ceiling QSPT performance (12.7 ± 0.5), suggesting a normative reference and confirming that pentagon copying in healthy ageing is well-executed when global cognition is preserved. The significant reduction observed across all patient groups underscores the sensitivity of the QSPT to neurodegenerative disease regardless of aetiology, with a graded impairment pattern from HC to PD to PSP/MSA/CBD. The ROC analysis for HC vs all parkinsonian patients (AUC=0.759, cutoff ≤12) complements the PD vs APS discrimination (AUC=0.764, cutoff ≤8) and synucleinopathies *versus* tauopathies (AUC=0.804, cutoff ≤11), and considering the high specificity values obtained, QSPT could be proposed as a screening single-item tool in the differentiation between parkinsonian syndrome. However, when QSPT score is higher than cut-off it would be useful to obtain other markers/clinical features to increase the accuracy in the differential diagnosis. Accurate early differentiation of PD from APS remains a major unmet clinical need, given that clinical diagnostic accuracy for PD is estimated at approximately 80% even in specialist settings, with the highest misdiagnosis rates occurring within the first two years of disease onset [22]. Thus, the high overlap in motor phenomenology between PD, PSP, MSA, and CBD—particularly in early or atypical phenotypes—makes rapid, low-cost ancillary markers highly desirable [23].

The pattern of QSPT scores across diagnostic groups could be associated with neuropathological substrates. CBD patients obtained the lowest score, consistent with the prominent visuoconstructive and ideomotor apraxia, according to parieto-frontal cortical degeneration observed in this condition [24]. PSP patients also showed significantly reduced QSPT compared to PD, with prominent rotation errors, potentially reflecting oculomotor dysfunction and impaired spatial reference frame processing, especially observed in PSP-Richardson’s syndrome [25, 26], both hallmarks of PSP. Considering MSA patients, they showed intermediate performances, although with high intra-group variability, consistent with the relative cognitive sparing reported in this condition [27]. Additionally, pentagon copying is known to be abnormal in a subset of patients with PD and predictive of later occurring dementia [28], thus even if PD patients obtained higher QSPT score than APS, within-group variability in PD could reflect patients in different stages of their disease.

Considering sub-item analysis, rotation and number-of-angles were the most discriminating parameters, which is broadly consistent with the broader QSPT literature documenting specific scoring profiles across neurodegenerative conditions [13, 14]. Notably, the sub-item “closing-in” showed significant group differences between PD and the whole APS group, especially with CBD patients. This phenomenon could be attributed to the loss of spatial inhibition arising from posterior parietal cortical degeneration [24].

Another interesting finding of the study is the association between QSPT and MRI morphometry. The negative correlation with MRPI could suggest that pentagon performance could be compatible with progressive brainstem neurodegeneration suggesting a neuroanatomical substrate for the observed cognitive-neuroimaging convergence. Particularly, previous studies on isolated brainstem lesions (e.g., due to stroke) indicates that damage to this brain area can lead to cognitive changes, including impairments in visuospatial processing, attention, and executive function [29]. Additionally, fMRI paradigm focused on the brainstem and cerebellum activation demonstrated that pons and midbrain are critical for the integration of visual information and visuospatial function [30]. These data suggest the brainstem acts as a key "activating" hub within the broader cognitive networks of the brain.

Regarding cognitive function, the correlation between QSPT and TMT-A is noteworthy. TMT-A primarily measures processing speed and visual scanning, functions mediated by fronto-striatal and parietal networks [31]. The correlation of QSPT performances with processing speed test across parkinsonian syndromes suggests that both tasks could rely on a common substrate of cortical-subcortical network integrity, mainly affected in APS patients.

Some limitations should be acknowledged. First, diagnoses were clinical rather than neuropathologically confirmed, but patients have been enrolled according to international diagnostic criteria. Second, APS subgroups were small, especially CBD patients, limiting power for within-APS comparisons and, thus we should consider our data exploratory rather than confirmatory. Additionally, the phenotypic heterogeneity within APS may contribute to intra-group score variability. Third, HC were not formally matched for education level, which may influence pentagon drawing performance; however, age and MMSE were comparable to the patient cohort. Fourth, due to the retrospective nature of the study, MRI analysis was limited to brainstem morphometry, which does not capture posterior parietal or frontal cortical atrophy, regions most directly implicated in visuoconstructive function. Future studies incorporating voxel-based morphometry or cortical thickness analysis will be needed to fully characterise the structural substrates of QSPT performance. At the same time, a comprehensive neuropsychological evaluation was not available for all patients, then the association between QSPT with other cognitive domains was not explored. Finally, it is important to acknowledge that, even if we obtained high specificity values in the AUC analysis, the low sensitivity values at both cutoff (65% for HC vs parkinsonism; 49% for PD vs APS) and the distributions overlap at the individual level across groups, could limit the use of QSPT especially when we obtain higher values than cut-off, thus requiring other markers for the differential diagnosis.

In conclusion, the quantitative MMSE pentagon score is a simple, zero-cost bedside tool that significantly discriminates PD from atypical parkinsonian syndromes. Its associations with MRI morphometry and processing speed could suggest a structural abnormalities involving cortico-subcortical networks. Prospective validation in early-stage parkinsonian cohorts and longitudinal studies are needed to establish its role in differential diagnostic pathways.

## Supplementary Information

Below is the link to the electronic supplementary material.

Supplementary file1 (DOCX 21 KB)

Supplementary file2 (DOCX 238 KB)

Supplementary file3 (DOCX 94 KB)

## Data Availability

The datasets generated and/or analysed during the current study are not publicly available due to privacy and ethical restrictions but are available from the corresponding author on reasonable request.
