# Supplementary material for: Interlocking pentagons task and visuospatial deficit in parkinsonian syndrome: a quantitative approach
Source: J Neurol. 2026 Sep 28;273(10):628. doi: 10.1007/s00415-026-14163-8 (PMC13619795; doi:10.1007/s00415-026-14163-8)
Supplement: Supplementary file 1 [file 415_2026_14163_MOESM1_ESM.docx]

**Table S1. QSPT total score and sub-items differences between patients groups**

| **QSPT Sub-item** | **Group 1** | **Group 2** | **p-value** |
| --- | --- | --- | --- |
| **QSPT total (0–13)** | **PD** | **PSP** | **<0.001** |
|  | **PD** | **MSA** | **0.040** |
|  | **PD** | **CBD** | **<0.001** |
|  | **PSP** | **MSA** | **0.042** |
|  | **PSP** | **CBD** | 1.000 |
|  | **MSA** | **CBD** | 0.085 |
| **Number of angles (0–4)** | **PD** | **PSP** | **<0.001** |
|  | **PD** | **MSA** | 0.077 |
|  | **PD** | **CBD** | **0.015** |
|  | **PSP** | **MSA** | 0.148 |
|  | **PSP** | **CBD** | 0.965 |
|  | **MSA** | **CBD** | 0.331 |
| **Intersection accuracy (0–4)** | **PD** | **PSP** | **<0.001** |
|  | **PD** | **MSA** | 0.192 |
|  | **PD** | **CBD** | **0.003** |
|  | **PSP** | **MSA** | **0.027** |
|  | **PSP** | **CBD** | 1.000 |
|  | **MSA** | **CBD** | **0.072** |
| **Open closure (0–2)** | **PD** | **PSP** | 0.183 |
|  | **PD** | **MSA** | 0.342 |
|  | **PD** | **CBD** | **0.002** |
|  | **PSP** | **MSA** | 0.849 |
|  | **PSP** | **CBD** | 0.206 |
|  | **MSA** | **CBD** | 0.158 |
| **Rotation (0–2)** | **PD** | **PSP** | **<0.001** |
|  | **PD** | **MSA** | 0.061 |
|  | **PD** | **CBD** | **0.003** |
|  | **PSP** | **MSA** | 0.097 |
|  | **PSP** | **CBD** | 0.789 |
|  | **MSA** | **CBD** | 0.258 |
| **Closing-in (0–1)** | **PD** | **PSP** | **0.007** |
|  | **PD** | **MSA** | 0.157 |
|  | **PD** | **CBD** | **0.007** |
|  | **PSP** | **MSA** | 0.625 |
|  | **PSP** | **CBD** | 0.604 |
|  | **MSA** | **CBD** | 0.267 |

Data are Mann-Whitney U statistic for continuous sub-items (number of angles, intersection accuracy, open closure, rotation) and Fisher's exact test for the closing-in sub-item. Abbreviations: PD, Parkinson's disease; PSP, progressive supranuclear palsy; MSA, multiple system atrophy; CBD, corticobasal degeneration; QSPT, Qualitative Scoring Pentagon Test; MMSE, Mini-Mental State Examination.
