# Supplementary material for: Interlocking pentagons task and visuospatial deficit in parkinsonian syndrome: a quantitative approach
Source: J Neurol. 2026 Sep 28;273(10):628. doi: 10.1007/s00415-026-14163-8 (PMC13619795; doi:10.1007/s00415-026-14163-8)
Supplement: Supplementary file 2 [file 415_2026_14163_MOESM2_ESM.docx]

**Interlocking pentagons task and visuospatial deficit in parkinsonian syndrome: a quantitative approach.**

Giulia Donzuso^1-2^, F. Cilio^1^, E. Ferrante^2^, C. D’Agate^3^, G. Fazzina^1^, C.E. Cicero^1^, G. Mostile^1-2^, A. Nicoletti^1^, M. Zappia^1^

^1^Department of Medical, Surgical Sciences and Advanced Technologies “G.F. Ingrassia”, University of Catania, Via Santa Sofia 78, 95123 Catania. Italy

^2^Oasi Research Institute - IRCCS, Troina, Italy.

^3^AOU Policlinico “G. Rodolico-San Marco”, Catania. Italy

**Corresponding author:** Professor Mario Zappia, Department of Medical, Surgical Sciences and Advanced Technologies 'G.F. Ingrassia', University of Catania, Via Santa Sofia 78, 95123 Catania, Italy. E-mail: m.zappia@unict.it

**Running title:** Interlocking pentagons in PD and APS

**Figure S1 Mean QSPT sub-item scores by diagnostic group (patients only).**

Bar heights represent group means ± SEM for continuous sub-items; the closing-in panel shows percentage of patients with closing-in present (Wilson 95% CI). *p<0.05; **p<0.01; ***p<0.001. Abbreviations: PD, Parkinson's disease; PSP, progressive supranuclear palsy; MSA, multiple system atrophy; CBD, corticobasal degeneration
